# Implementation Fidelity of Accredited Social Health Activist (ASHA) Workers in Malaria Case Management in a High-Burden District of North India

**DOI:** 10.7759/cureus.96103

**Published:** 2025-11-04

**Authors:** Ezhilarasan Selvaraju, Pawan K Goel, Neeraj Gour, Arun Kumar

**Affiliations:** 1 Community Medicine, Bhaarath Medical College and Hospital, BIHER (Bharath Institute of Higher Education and Research) University, Chennai, IND; 2 Community Medicine, Shaheed Hasan Khan Mewati Government Medical College, Nalhar, IND; 3 Preventive Medicine, Shaheed Hasan Khan Mewati Government Medical College, Nalhar, IND

**Keywords:** community health volunteers, fever, fidelity implementation outcome, implementation research (ir), malaria, under-five children

## Abstract

Background

Strengthening the fidelity of community-based fever case management remains pivotal to reducing malaria morbidity and achieving India’s goal of malaria elimination by 2030.

Objective

To assess the implementation fidelity of accredited social health activist (ASHA) workers in malaria case management, including diagnosis, treatment, and follow-up, in a high-burden district of North India.

Methods

A community-based cross-sectional study was conducted from May to September 2019 in the catchment area of PHC Ujina, Nuh, Haryana, India. Using multi-stage random sampling, 440 mothers of under-five children and 30 ASHAs were interviewed with pre-tested tools. Implementation fidelity was assessed across four WHO-aligned domains: reach, treatment initiation, treatment completion, and follow-up. Data were analyzed in Epi Info™ version 7.2.2.16 (Centers for Disease Control and Prevention, Atlanta, USA), with programme benchmarks tested using Z and exact binomial tests.

Results

Half of the under-five children (222/440, 50.5%) experienced fever in the preceding month. Among them, only four (1.8%) mothers sought care first from ASHAs, significantly below the benchmark of 80% (p < 0.05). Malaria testing was delayed in most cases; only nine children (4.0%) were tested within 24 hours of fever onset. Among malaria-positive cases (n = 8), ASHA fidelity was high: treatment initiation and completion were achieved in all cases (100%), and follow-up was conducted in seven children (87.5%). While maternal knowledge of transmission was high (92.1% identified mosquito bites), three-fourths (73.6%) still preferred informal providers such as Hakims and traditional healers for fever management.

Conclusion

ASHAs demonstrated strong adherence to malaria case management protocols, but remain underutilized as the first point of care. Strengthening community engagement, ensuring rapid diagnostic access, and enhancing supportive supervision are critical to improving ASHA reach and accelerating India’s malaria elimination agenda.

## Introduction

Malaria remains one of the world’s most persistent public health concerns, with 263 million cases and 597,000 deaths reported across 83 endemic countries in 2023 [1]. The World Health Organization (WHO) stresses the importance of universal access to preventive tools, early diagnosis, and prompt treatment to reduce morbidity and mortality [2]. In endemic regions, fever is the most frequent presenting symptom of malaria, and its timely recognition and management are critical to preventing progression to severe disease [1].

India has committed itself to malaria elimination in line with the WHO Global Technical Strategy 2016-2030 and the Asia Pacific Leaders Malaria Alliance Roadmap [3]. The National Framework for Malaria Elimination (2016-2030) aims to interrupt indigenous transmission by 2030 while ensuring that malaria-free areas remain protected [3]. Yet, national achievements mask regional disparities. In 2018, the Annual Parasite Incidence (API) of Haryana was 0.11 and the national API was 0.32, but Nuh district reported an API of 1.42-over 12 times the state average-indicating a concentrated burden in this district [4].

Community health workers are central to malaria case management in resource-limited settings [5]. In India, Accredited Social Health Activist (ASHA) workers serve as the key link between households and the health system under the National Vector Borne Disease Control Programme (NVBDCP). Their responsibilities include performing rapid diagnostic tests (RDTs) or preparing smears for microscopy, initiating treatment for confirmed cases, ensuring treatment completion through follow-up, promoting the use of insecticide-treated nets, and mobilizing households for indoor residual spraying. Severe or complicated cases are referred promptly to higher facilities as per national guidelines [3]. Strengthening ASHA-led fever case management is therefore essential for meeting India’s malaria elimination targets.

Beyond the availability of services, the fidelity of implementation, that is, how closely health workers deliver interventions as originally intended-determines whether programmes achieve their expected outcomes [6]. Even with adequate training and supplies, weak fidelity can undermine impact. While previous studies have examined the roles of ASHA in mobilization and service delivery, limited evidence exists on how faithfully they adhere to malaria case management protocols, especially in high-burden districts.

Effective malaria case management requires that febrile children are tested within 24 hours, confirmed cases are treated with nationally recommended drugs at correct doses, and adherence is monitored through follow-up [3]. Assessing whether ASHAs deliver these steps with fidelity is critical to closing the gap between programme design and real-world outcomes.

Given the persistently high malaria transmission in Nuh district, Haryana, this study applies an implementation fidelity framework to evaluate the performance of ASHAs in the management of childhood fever and malaria case detection.

Objective

To assess the implementation fidelity of ASHAs in malaria case management, including diagnosis, treatment, and follow-up, in a high-burden district of North India.

## Materials and methods

Study area and population

The study was conducted in Nuh district (formerly Mewat) of southern Haryana, India. Nuh is one of the most socioeconomically disadvantaged districts in the country, with a projected population of 1.57 million in 2025, compared to 1.09 million in 2011 [7]. Nearly 89% of the population resides in rural areas, with an overall literacy rate of 54.1% and female literacy only 36.6% in Nuh district. The sex ratio (907 females per 1000 males) and child sex ratio (906) are below national averages, reflecting entrenched gender disparities. Approximately one-quarter of the population is below six years of age, making the district particularly vulnerable to childhood infectious diseases [7].

High population density (723 persons/km² in 2011), overcrowding, poor sanitation, and dependence on informal healthcare providers compound the health challenges. Extensive agriculture and water stagnation provide favorable conditions for vector breeding, sustaining malaria transmission. Entomological surveys in the area have consistently identified *Anopheles culicifacies* (species A) as the dominant malaria vector, with peak activity during the monsoon season [8].

Administratively, Nuh comprises four tehsils (Taoru, Nuh, Ferozepur Jhirka, and Punhana), served by one district hospital, one medical college hospital, and four community health centers. Despite these facilities, more than three-fourths of the district’s malaria cases in 2018 originated from the catchment area of PHC Ujina under CHC Nuh (Office of the Civil Surgeon, Nuh). Therefore, villages under PHC Ujina were selected for the present study.

Study design and period

A community-based cross-sectional study was carried out between May and September 2019, after the introduction of ASHA-led fever case management activities in the study area.

Intervention context: role of ASHAs 

In India’s malaria elimination strategy [3], ASHAs serve as the first point of contact at the community level. Their responsibilities include health education, promotion of preventive measures such as long-lasting insecticidal nets (LLINs), and case management of febrile illnesses. Under the NVBDCP, ASHAs are trained to use bivalent rapid diagnostic tests (RDTs), collect blood smears for microscopy, provide treatment according to national drug policy for *Plasmodium vivax* and *Plasmodium falciparum*, identify severe malaria cases for referral, and ensure adherence through follow-up visits.

Their work is supervised by multipurpose health workers (MPHWs), auxiliary nurse midwives (ANMs), and medical officers at PHCs. Malaria technical supervisors and district vector-borne disease officers conduct random verifications and provide supportive supervision. ASHAs also maintain fever registers and submit reports for consolidation at PHC, district, and state levels.

Study participants

The study population comprised (i) mothers of under-five children residing in the catchment area of PHC Ujina for at least six months, and (ii) all ASHAs working in the study villages, irrespective of years of service. Mothers unwilling to participate or those with under-five children having major comorbidities were excluded.

Sample size and sampling

The sample size was estimated to assess the implementation fidelity of ASHAs in managing childhood fever. Since no prior estimates of appropriate and timely fever management by ASHAs were available from Nuh district, a prevalence (P) of 50% was assumed to yield the maximum sample size. Using the single-proportion formula $n = \frac{Z^2 P Q}{d^2}$, with a 95% confidence level (Z = 1.96) and 5% absolute precision (d = 0.05), the minimum required sample size was calculated as 384. To account for a potential 15% non-response rate, the final target sample was set at 440 mothers of under-five children.

The sampling techniques used are multi-stage random sampling. The sample was proportionally distributed across all four sub-centres under PHC Ujina, an area contributing over three-fourths of the district’s malaria burden. From each sub-centre, 110 mothers were selected. Within each sub-centre, two ASHA catchment areas were randomly chosen by lottery, and 55 eligible mothers from each ASHA area were included through house-to-house visits. If a respondent was unavailable after three visits or declined participation, the nearest eligible mother was enrolled. This ensured representativeness across ASHA coverage areas while maintaining the required sample size.

Operational definitions of fidelity indicators

ASHA Reach

Proportion of fever cases in under-five children for whom caregivers first sought care from an ASHA.

Treatment Initiation

Proportion of malaria-positive under-five children for whom ASHA initiated or facilitated antimalarial treatment on the same day as diagnosis.

Treatment Completion

Proportion of malaria-positive under-five children who completed the full antimalarial course under ASHA supervision.

Follow-Up

Proportion of malaria-positive under-five children for whom ASHA conducted follow-up visits to ensure adherence, symptom resolution, or referral if needed.

Data collection

Data were gathered using a pre-tested, semi-structured interview schedule administered in Hindi. Mothers were asked about sociodemographic details, fever episodes in the preceding month, care-seeking behavior, diagnostic testing, treatment received, and involvement of ASHAs. A separate tool assessed ASHAs’ knowledge, practices, and training experience.

Socioeconomic status was classified using the Modified BG Prasad Scale [9], applying the consumer price index-industrial workers (CPI-IW) value of 314 for May 2019.

Data analysis

Epi Info™ version 7.2.2.16 (Centers for Disease Control and Prevention, Atlanta, USA) was used for data entry and statistical analysis. Frequencies, percentages, and medians were computed for descriptive statistics. Fidelity indicators were expressed as proportions against programme benchmarks. One-sample tests of proportions were applied: the Z-test was used for indicators with large denominators, and the exact binomial test was used for indicators with small denominators (n < 30). A p-value < 0.05 was considered statistically significant. Findings were compared against the WHO and NVBDCP programme benchmarks to assess fidelity gaps in relation to the study objectives.

Ethical considerations

Approval was obtained from the Institutional Ethics Committee, Shaheed Hasan Khan Mewati (SHKM) Government Medical College (GMC), Nalhar, Nuh (Ref: SHKM/IEC/2019/98, dated May 2, 2019). Permission was secured from the Medical Officer, PHC Ujina. Written informed consent was taken from all participants. Confidentiality, voluntary participation, and the right to withdraw were assured. Children found febrile during the survey were referred for testing and treatment under NVBDCP guidelines.

## Results

ASHAs (service providers)

Characteristics of ASHAs

Thirty ASHAs from four sub-centres under PHC Ujina were interviewed. The median age was 35 years (IQR: 30-39). Most ASHA (19; 63.3%) had studied up to primary or middle school, while only 9 ASHA (30.0%) had completed matriculation or above.

Knowledge of Malaria and Fever Case Management

All ASHAs recognized malaria as a major community health concern and a frequent cause of fever. Nearly all (29; 96.7%) knew mosquito bites as the mode of transmission, but only six (20.0%) were aware of nocturnal biting behavior. Pregnant women were widely identified as at-risk (26; 86.7%), while fewer mentioned under-five children (4; 13.3%). Fever was universally recognized as the key symptom, and severe malaria was most often associated with unconsciousness (22; 73.3%) and high fever (17; 56.6%). Almost all (29; 96.7%) reported having received formal training in malaria prevention and case management

Practices in Fever Case Management

All ASHAs reported managing childhood fever cases, with most (43.3%) attending to 6-10 cases monthly. Nearly all (29; 96.7%) performed rapid diagnostic tests (RDTs), though only 16 (53.3%) were aware of microscopy for confirmation. All RDT-positive cases were treated on the same day (100%), and compliance was universally checked until treatment completion. Non-responding children were referred to higher facilities. For vomiting of tablets, responses varied-16 (53.3%) advised re-administering after food, while others recommended repeating immediately or after a short interval.

Mothers of under-five children

Characteristics of Mothers

A total of 440 mothers participated. Their median age was 26 years (IQR: 23-32). The largest age group was 21-25 years (158; 35.9%), followed by 26-30 years (135; 30.7%). Most respondents were Muslim (329; 74.8%) and illiterate (293; 66.6%), while nearly all (439; 99.8%) were homemakers. Nuclear families accounted for 330 households (75.0%), with an average family size of 6.9. Based on the modified BG Prasad classification (CPI May 2019), 275 (62.5%) families belonged to the lower-middle socioeconomic class.

Knowledge of Malaria Transmission, Symptoms, and Treatment 

Most mothers (405; 92.1%) identified mosquito bites as the route of transmission of malaria, and an equal proportion (405; 92.1%) were aware of seasonal variation. Awareness of evening/night biting was lower (243; 55.2%). Fever was the most commonly cited symptom (305; 69.3%), followed by headache (152; 34.6%) and fatigue (85; 19.3%). A total of 108 mothers (24.5%) could not name any symptoms of severe malaria. Blood testing was reported as the main diagnostic method by 374 (85.0%). For treatment, 293 (66.6%) preferred PHC/CHC facilities, but 211 (47.9%) also reported consulting informal providers (Table 1).

**Table 1:** Knowledge about transmission, symptoms, and treatment of malaria among mothers (n = 440) * Multiple responses allowed PHC: primary health center; CHC: community health center

Variable	Category	Frequency (n)	Percentage (%)
Transmission	Mosquito bite	405	92.1
	Evening/night biting habit	243	55.2
	Awareness of seasonal variation	405	92.1
Symptoms of malaria*	Fever	305	69.3
	Headache	152	34.6
	Fatigue	85	19.3
Severe malaria symptoms	Could not identify any	108	24.5
Diagnosis	Blood test	374	85.0
Treatment centre	PHC/CHC	293	66.6
	Traditional healer	211	47.9

Care-Seeking Attitude

When asked about preferred care-seeking during a child’s fever, 324 mothers (73.6%) stated that they usually approached informal providers (Hakims/traditional healers). A slightly smaller proportion (295; 67.1%) reported using government hospitals. Other common practices included home remedies (132; 30.0%) and visits to medical shops (99; 22.5%). Nearly all mothers (435; 98.9%) stated that the child’s condition was the main determinant of seeking care, while only 5 (1.1%) mentioned cost (Table 2)

**Table 2:** Care-seeking attitudes of mothers of under-five children for childhood fever (n = 440) * Multiple responses allowed

Variables	Frequency	Percentage
What mothers would mothers do when their child has fever*		
Go to hospital (government)	295	67.1
Self-treat at home	132	30.0
Go to medical shop	99	22.5
Go to local traditional healer	324	73.6
Important factor which mothers would consider when seeking care for their sick child with fever		
Condition of child	435	98.9
Perceived cost of treatment	5	1.1

First Point of Contact for Fever Episodes

Out of 440 mothers, 222 (50.5%) reported at least one fever episode in their under-five children during the previous month. Among these, 211 (93.7%) sought treatment, while 11 (4.9%) did not. The majority first consulted informal providers (Hakims/traditional healers: 160; 76.0%). Only 26 (12.0%) went directly to a PHC/CHC, while very few approached ANMs (8; 4.0%), ASHAs (4; 1.8%), or government hospitals/medical colleges (10; 5.0%).

Promptness of Malaria Testing in Febrile Cases

Among 222 children with fever, 125 (56.0%) were not tested for malaria. Only nine (4.0%) were tested on the same day of fever onset, while 50 (23.0%) were tested after two days, six (3.0%) after three days, and 24 (11.0%) after more than three days. These results reveal significant diagnostic delays, with most children not meeting the national recommendation for parasitological confirmation within 24 hours.

ASHA case management: high fidelity but low reach

ASHA implementation fidelity showed a dual picture. Among 222 febrile children, only four (1.8%) consulted ASHAs as their first point of care, reflecting extremely low reach. In contrast, among malaria-positive children (n = 8), ASHAs demonstrated high fidelity: all (100%) received treatment on the same day, completed the full prescribed antimalarial course, and 7 (87.5%) were followed up to ensure adherence.

When compared with programme benchmarks, ASHA reach (1.8%) was significantly below the target of 80% (p < 0.0001). However, treatment initiation and treatment completion both met the 100% benchmark (p = 1.0). Follow-up was achieved in 87.5% of cases, which, though slightly below the 100% benchmark, was not statistically different (Table 3).

**Table 3:** Fidelity of ASHA performance in malaria case management against programme targets * Z-test for one sample proportion was applied for ASHA reach, while the exact binomial test was used for ASHA treatment initiation on the same day, treatment completion under follow-up, and fidelity of ASHA follow-up. ** Significant P value ASHA: accredited social health activist

Indicator	n	N	Proportion (%)	Statistical test	Test statistic	p-value*
ASHA reach – % of fever cases diagnosed and treated within 24 hrs by ASHA (target 80%)	4	222	1.8	Z test	Z = -29.2	<0.001**
ASHA treatment initiation same day (target 100%)	8	8	100	Binomial test	Exact (8/8)	1
Treatment completion under ASHA supervision (target 100%)	8	8	100	Binomial test	Exact (8/8)	1
Fidelity of follow-up (target 100%)	7	8	87.5	Binomial test	Exact (7/8)	0.289

## Discussion

This study examined maternal knowledge and care-seeking practices for childhood fever, alongside the fidelity of ASHA-led malaria case management, in a high-burden setting of Nuh district, Haryana. The findings reveal a paradox: ASHAs consistently adhered to malaria management protocols once cases reached them, yet their overall reach within the community was strikingly limited. Despite the presence of ASHAs in every village, nearly three-fourths of mothers preferred informal providers such as Hakims and traditional healers when seeking care for their children. This reliance was shaped by long-standing cultural trust in local healers, low literacy, limited awareness of ASHA services, and the convenience of informal providers. These demand-side barriers reduced the effective utilization of ASHAs, even though they demonstrated the capacity to provide timely and guideline-based malaria case management.

Knowledge about malaria transmission among mothers was high, with more than 90% correctly identifying mosquito bites as the source, which is comparable to findings from earlier studies in India [10]. Fever emerged as the most frequently recognized symptom (69.3%), a result in line with observations from Myanmar [11]. However, despite this awareness, mothers’ care-seeking behaviour reflected deep reliance on informal providers. Nearly three-fourths reported consulting traditional healers, a pattern that diverges from studies in Nigeria and Myanmar, where formal health facilities or community workers were more commonly sought for childhood fever [11,12]. In Nuh, the preference for informal providers is likely driven by cultural trust, accessibility, and affordability, which outweigh awareness of formal services.

Another key gap identified was delayed diagnostic testing. Among febrile children, more than half (56.0%) were not tested for malaria, and only 4.0% were tested within the recommended 24 hours of fever onset. Similar delays have been reported in Ethiopia [13], where perceptions of mild illness, long travel distances, and financial constraints contributed to missed or delayed testing. These findings underscore the ongoing challenge of aligning community practices with NVBDCP guidelines, which mandate parasitological confirmation within 24 hours of fever onset.

In contrast, once children were seen by ASHAs, fidelity to malaria management protocols was strong. All malaria-positive children in this study received same-day treatment, all completed the prescribed antimalarial regimen under supervision, and follow-up was ensured for most cases. These results resonate with studies from Cambodia [14] and Cameroon [15], where community health workers demonstrated high adherence to diagnostic and treatment standards when adequately trained and supervised. The mismatch between high technical fidelity and low community utilization highlights the need to address demand-side barriers rather than supply-side shortcomings.

Strengths

Conducted in a high-burden district, the study provides insights from a priority setting where malaria elimination efforts face the greatest challenges. A relatively large, representative sample of 440 mothers across all four sub-centres of PHC Ujina strengthened reliability and generalizability to the local context. Inclusion of both providers (ASHAs) and beneficiaries (mothers) offered a comprehensive perspective on implementation fidelity. Standardized, pre-tested tools enhanced the accuracy of data collection. Use of WHO-aligned fidelity indicators ensured comparability with international benchmarks.

Limitations

The cross-sectional design limited the ability to infer causality between ASHA involvement and child health outcomes. Maternal recall of fever episodes may have introduced recall bias. Stock-outs of RDTs or antimalarial drugs, which could affect diagnostic and treatment practices, were not assessed. Fathers and other caregivers, who may influence treatment decisions, were not included. Social desirability bias may have influenced self-reported practices from both ASHAs and mothers. Findings from a single PHC area may not be generalizable to districts with different health systems or socio-cultural contexts.

## Conclusions

ASHAs showed strong fidelity to malaria case management protocols, ensuring complete treatment and follow-up when cases reached them. Yet, their role as the first point of care remained limited due to low diagnostic access and reliance on informal providers. Strengthening community-level diagnostics, fostering trust in ASHAs, and creating referral linkages with informal providers can enhance early case detection and programme reach. These actions provide actionable evidence to guide policy refinement and accelerate India’s malaria elimination efforts.

## References

[1] (2025). Malaria. https://www.who.int/news-room/fact-sheets/detail/malaria.

[2] (2025). Global technical strategy for malaria 2016-2030, 2021 update. https://www.who.int/publications/i/item/9789240031357.

[3] (2025). Operational manual for malaria elimination in India (version 1). https://ncvbdc.mohfw.gov.in/WriteReadData/l892s/5232542721532941542.pdf.

[4] (2025). Epidemiological situation report of Andhra Pradesh 2018. https://ncvbdc.mohfw.gov.in/Doc/Annual-Report-2018.pdf.

[5] Smith Paintain L, Willey B, Kedenge S (2014). Community health workers and stand-alone or integrated case management of malaria: a systematic literature review. Am J Trop Med Hyg.

[6] Proctor E, Silmere H, Raghavan R (2011). Outcomes for implementation research: conceptual distinctions, measurement challenges, and research agenda. Adm Policy Ment Health.

[7] (2025). Mewat District Population Census 2011 - 2021 - 2025, Haryana literacy sex ratio and density [Internet]. [cited 2025 Sep 27]. Available from. https://www.census2011.co.in/census/district/226-mewat.html.

[8] Nanda N, Singh SP, Prajapati BK, Ranjan K, Kar NP, Sharma SK, Valecha N (2017). Entomological determinants of malaria transmission in an epidemic prone area of district nuh (Haryana state), India. J Vector Borne Dis.

[9] Pandey VK, Aggarwal P, Kakkar R (2019). Modified BG Prasad socio-economic classification, update - 2019. Indian J Community Health.

[10] Kimbi HK, Nkesa SB, Ndamukong-Nyanga JL, Sumbele IU, Atashili J, Atanga MB (2014). Knowledge and perceptions towards malaria prevention among vulnerable groups in the Buea Health District, Cameroon. BMC Public Health.

[11] Thandar MM, Kyaw MP, Jimba M, Yasuoka J (2015). Caregivers' treatment-seeking behaviour for children under age five in malaria-endemic areas of rural Myanmar: a cross-sectional study. Malar J.

[12] Adedotun AA, Morenikeji OA, Odaibo AB (2010). Knowledge, attitudes and practices about malaria in an urban community in south-western Nigeria. J Vector Borne Dis.

[13] Aderaw Z, Gedefaw M (2013). Knowledge, attitude and practice of the community towards malaria prevention and control options in anti-malaria association intervention zones of Amahara National Regional State, Ethiopia. J Trop Dis Public Health.

[14] Canavati SE, Lawpoolsri S, Quintero CE (2016). Village malaria worker performance key to the elimination of artemisinin-resistant malaria: a Western Cambodia health system assessment. Malar J.

[15] Shey DN, Jules Clement AN, Muluh N, Wung BA, Katte IK (2017). Community health workers’ knowledge, attitudes and practices regarding malaria control and prevention in Bamenda, Cameroon: a community based study. J Health Med Inform.

